# Capsular Exopolysaccharides from Two *Streptococcus thermophilus* Strains Differ in Their Moisture Sorption Behavior

**DOI:** 10.3390/foods12030596

**Published:** 2023-01-31

**Authors:** Carsten Nachtigall, Georg Surber, Daniel Wefers, Cordula Vogel, Harald Rohm, Doris Jaros

**Affiliations:** 1 Institute of Natural Materials Technology, Technische Universität Dresden, 01062 Dresden, Germany; 2 Institute of Chemistry, Food Chemistry–Functional Food, Martin Luther University Halle-Wittenberg, 06120 Halle (Saale), Germany; 3 Institute of Soil Science and Site Ecology, Technische Universität Dresden, 01062 Dresden, Germany

**Keywords:** capsular exopolysaccharide production, lactic acid bacteria, water binding capacity, structural composition, sorption kinetics, ropiness, exopolysaccharide isolation

## Abstract

*Streptococcus thermophilus* is a species frequently used in the manufacture of fermented milk. Apart from acid production, some strains additionally synthesize exopolysaccharides (EPS) which contribute to texture improvement and syneresis reduction, both being attributable to the EPS’s high water binding capacity. There are two different types of EPS that may be produced, namely free exopolysaccharides (fEPS) which are secreted into the medium, and capsular EPS (cEPS) which remain attached to the bacterial cell wall. This study aims to analyze their individual contribution to techno-functional properties of fermented milk by determining the moisture sorption behavior of isolated fEPS and cell-attached cEPS from two *S. thermophilus* strains separately: ST-1G, a producer of non-ropy fEPS and cEPS, and ST-2E, a producer of ropy fEPS and cEPS. Differences in moisture load and sorption kinetics, determined for the first time for microbial EPS, were related to structural and macromolecular properties. The observed data are discussed by using previously published data on the physical properties of stirred fermented milk produced with these two strains. ST-1G EPS showed a higher cEPS fraction, a higher moisture load and slower moisture desorption than EPS produced by ST-2E, thus contributing to lower syneresis in fermented milk. For ST-2E, higher gel viscosity was related to a higher intrinsic viscosity and molecular mass of the ropy fEPS. Both strains produced complex EPS or EPS mixtures with clearly different molecular structures.

## 1. Introduction

In fermented milk manufacture, the primary purpose of using lactic acid bacteria (LAB) as starter culture is the conversion of lactose to lactic acid. In addition to acidification, some LAB are able to produce exopolysaccharides (EPS) that, depending on the strain, differ in structural properties (e.g., monosaccharide composition and linkage, degree of branching and length of side chains) and macromolecular properties (e.g., molecular mass, intrinsic viscosity), but generally show a high water binding capacity [1,2]. *Streptococcus thermophilus* strains as important representatives of LAB synthesize extracellular hetero-polysaccharides. Most of the EPS are usually present in the serum phase of the fermented milk (free EPS, fEPS) and may be further classified by the degree of ropiness they induce in the product. Such ropiness can easily be assessed by pulling a spoon out of the product and judging the appearance of the resulting thread [1]. In contrast, some EPS remain attached to the bacterial cell walls (capsular EPS, cEPS). These cEPS can be visualized by negative staining with Indian ink and light microscopy [3], or by using confocal laser-scanning microscopy in reflectance mode [4]. The capsule size was found to be affected by the carbon and nitrogen sources present in the fermentation media, and larger capsules may indicate a higher cEPS amount [5]. Due to the low amount and difficult isolation, only limited information is available on structural and macromolecular properties of capsular EPS.

The techno-functional impact of EPS produced in situ by *S. thermophilus* during fermentation of milk (e.g., enhanced viscosity and ropiness and/or lower susceptibility to ‘wheying-off’ = syneresis) is, however, still not fully understood, but has frequently been attributed to the type of EPS. Ropy fEPS were reported to contribute to creaminess and viscosity, while non-ropy fEPS may also increase viscosity [1,2]. Syneresis of fermented milk was often lower with cEPS producers especially when large capsules were present [6,7,8]. In situ studies, however, have the drawback that observations cannot be attributed to a specific EPS type due to the presence of both fEPS and cEPS.

Syneresis phenomena in fermented milk may be linked to moisture adsorption and desorption properties of in situ produced EPS. After isolation of pure EPS, they may be individually subjected to dynamic vapor sorption experiments for analyzing their water binding behavior. Recent studies revealed a similar moisture load of *S. thermophilus* ST-143+ cells with cEPS and fEPS produced by this strain, which was significantly higher than that of cells from which the cEPS were removed [3]. Different equilibrium sorption models with a validity in a specific relative humidity (*r.h.*) range (e.g., Brunauer–Emmett–Teller (BET), Guggenheim–Anderson–De Boer (GAB)) are commonly applied to interpret moisture adsorption and desorption isotherms of foods [9,10]. However, the determination of sorption kinetics is not standardized yet. Especially for moisture adsorption of plant-based compounds (e.g., cellulose, wood fiber, durum wheat), different kinetic models have been introduced [11], including the Fickian diffusion model [12,13], the swelling stress model [14,15], the thermally limited moisture transport model [16] and the parallel exponential kinetics model [17,18]. The latter was also applied to different foods such as peas, garlic and cereals [19,20,21] but, to our knowledge, no studies exist for the sorption kinetics of bacterial EPS so far. General criticism as concerns the applied models arises as none of the mentioned models describes sorption phenomena sufficiently, and concerns were also raised as adsorption or desorption experiments are often aborted without reaching equilibrium, resulting in erroneous mathematical fits and interpretations [22].

For stirred fermented milk in particular, the EPS techno-functionality depends on the complex interplay between intrinsic factors (e.g., amount and properties of EPS) and several extrinsic factors (e.g., base milk composition, temperature, shearing). For the identification of key parameters, we previously discriminated the effects of intrinsic factors (2 EPS producers and a reference strain) and extrinsic factors (3 process parameters) on physical properties of stirred gels by multivariate ANOVA-simultaneous component analysis [23]. We concluded that EPS from ST-2E mainly determine texture properties, whereas the extrinsic factors primarily affect acidification kinetics and EPS amount. EPS from ST-1G reduced syneresis, and this effect was more pronounced when the fraction of whey proteins in the base milk was increased. The total EPS amount did not correlate with texture properties or syneresis, pointing to the importance of structural and physical features for their techno-functional impact on stirred fermented milk. As ST-1G and ST-2E were both classified as cEPS producers, but differed in their fEPS type (ST-2E: ropy fEPS; ST-1G: non-ropy fEPS), it was not possible to attribute the results to one specific EPS type. This study therefore aims to analyze the moisture sorption behavior of cells with attached cEPS and the respective pure fEPS from ST-1G and ST-2E after in situ production and subsequent isolation. To explain differences in moisture load and sorption velocity, which was analyzed for the first time for microbial EPS, structural and macromolecular EPS properties were studied and related to the physical properties of stirred gels.

## 2. Materials and Methods

### 2.1. Materials

*S. thermophilus* strains ST-1G and ST-2E were provided by two starter culture producers, and whey permeate powder was from Wheyco GmbH (Altentreptow, Germany). All chemicals were obtained from Carl Roth GmbH & Co. KG (Karlsruhe, Germany) and Merck KGaA (Darmstadt, Germany).

### 2.2. Cultivation Conditions 

*S. thermophilus* cultivations were carried out in a 5 L bioreactor (Applikon^®^ Biotechnology BV, Delft, The Netherlands) according to Nachtigall et al. [24]. In brief, solutions of 100 g/L whey permeate powder, 34.2 g/L lactose, 9 g/L glucose, 10 g/L tryptone and 2 g/L ammonium sulphate in deionized water were inoculated with 50 mL pre-culture of ST-1G or ST-2E, and incubated anaerobically at 40 °C while stirring of 200 rpm. The pH was maintained at 6.0 through automatic addition of NaOH. The cultivation was stopped at the end of the exponential growth phase, and the medium was stored at −80 °C until further use.

Stirred skim milk gels were manufactured according to Surber et al. [25] by inoculation of 450 mL skim milk (100 g/kg dry matter) with 4.5 mL pre-culture and fermentation until pH 4.60 at 40 °C, followed by stirring and storage at 6 °C.

### 2.3. Biomass Determination and Exopolysaccharide Quantification

For each strain, 10 mL of cultivated medium was centrifuged (19,000× *g*, 15 min, 6 °C) in triplicate, the supernatant was removed, and the remaining pellet was washed twice with 40 mL deionized water and centrifuged again. After freeze-drying of the pellet (Alpha 1–2, Martin Christ Gefriertrocknungsanlagen, Osterode am Harz, Germany) its mass, representing cell biomass *X* [g/kg], was determined using a balance.

EPS were isolated for subsequent quantification in analytical scale (5 mL) as described in detail by Mende et al. [8]. After centrifugation of the fermented medium (19,000× *g*, 15 min, 6 °C) to remove cells, 5 mL of the supernatant was incubated with 250 µL of 4.8 mg/mL Pronase E solution (pH 7.5, 37 °C, 24 h). After adding 0.7 mL of 800 g/L trichloroacetic acid, the precipitated proteins were removed by centrifugation (19,000× *g*, 15 min, 6 °C). The EPS in the supernatant were then precipitated with two volume units of cold acetone, separated by centrifugation (conditions as above) and resuspended in deionized water. The resulting EPS solution was dialyzed against deionized water (molecular weight cut-off: 6–8 kDa, Carl Roth GmbH & Co. KG, Karlsruhe, Germany) and then freeze-dried. This procedure resulted in fEPS. To isolate the total amounts of EPS (tEPS = sum of fEPS + cEPS), an initial heating step (90 °C, 10 min) was applied as first step and prior to the cell removal to release cEPS into the surrounding medium. EPS were quantified photometrically using the method of Dubois et al. [26]. fEPS and tEPS concentrations are expressed as mg glucose equivalents/L (mg GE/L), and the amount of cEPS was derived as the difference between tEPS and fEPS. The yields *Y*_EPS_/*X* [mg/g] and *Y*_cEPS_/*X* [mg/g] were calculated by relating EPS amounts to biomass.

### 2.4. Visualization of Capsular Exopolysaccharides

Stirred skim milk gel was diluted 1:1 with deionized water. Then, 5 µL of the solution was mixed with 5 µL India ink on a glass slide and covered with a cover slip. Microscopic images were taken at 1000-fold magnification. cEPS occur as white zones around the cells in microscopic images.

For SEM imaging, 2 mL cell suspensions of *S. thermophilus* ST-1G were centrifuged (14,100× *g*, 5 min, room temperature) and washed three times with 9.0 g/L sodium chloride solution, and finally with deionized water, to remove remnants of culture media. The remaining cell pellets were at first fixated in 40 g/L paraformaldehyde with 2 mL glutaraldehyde in 100 mL 0.1 mol/L sodium cacodylate buffer for 45 min. Subsequently, the samples were washed three times with 0.1 mol/L sodium cacodylate buffer for 10 min and once in deionized water. As the next step, samples were stained with 5 g/L RuO_4_ for 60 min and residual RuO_4_ was removed by five further washing steps with deionized water. Afterwards, samples were freeze-dried and fastened on aluminum specimen holders using conductive double-sided adhesive carbon tabs and coated with approximately 40 nm carbon, using an EMITECH K905 Carbon Coater (Emitech Ltd., Ashford, Kent, England). Bacterial cells were visualized with an environmental scanning electron microscope under high vacuum (Quanta FEG 650, Thermo Fisher Scientific, Waltham, MA, USA) at 10 kV beam energy and a spot size of 2.5.

### 2.5. Preparation of Cell and Exopolysaccharide Isolates

For further analyses, cell and fEPS isolates were obtained by crossflow filtration (Sartorius Stedim Biotech GmbH, Göttingen, Germany) as described by Nachtigall et al. [27]. Briefly, 0.1 µm polyether sulfone microfiltration membranes were used to separate cells from fEPS and 5 kDa polyether sulfone ultrafiltration membranes to purify cell and fEPS isolates. fEPS were subsequently precipitated from the 5 kDa retentate by adding two volume units of cooled acetone. After 24 h at 6 °C, the EPS pellet was dissolved in deionized water. Finally, all isolates were freeze-dried and stored in a desiccator until further use.

### 2.6. Structural Composition of Free Exopolysaccharides

The monosaccharide composition of fEPS was determined by HPAEC-PAD after acid hydrolysis. Briefly, 2 mol/L TFA was added to the fEPS and hydrolysis was carried out for 1 h at 121 °C. After evaporation of the acid, monosaccharides were dissolved in ultrapure water and analyzed on an ICS-5000 HPAEC-PAD system (Thermo Scientific Dionex, Sunnyvale, CA, USA) equipped with a CarboPac PA20 column. Detailed separation conditions were described by Fels et al. [28]. Methylation analysis was used to determine the type and abundance of glycosidic linkages. In brief, the samples were dissolved in DMSO and successively incubated with powdered sodium hydroxide and methyl iodide. Methylated polysaccharides were extracted into dichloromethane, dried, and hydrolyzed by 2 mol/L TFA for 1.5 h at 121 °C. Subsequently, partially methylated monosaccharides were reduced by using NaBD_4_ and acetylated by using 1-methylimidazole and acetic anhydride. After extraction, the partially methylated alditol acetates (PMAAs) were identified by GC-MS. The PMAA portions were estimated by using GC-FID. A detailed description of the procedure can be found elsewhere [28]. For the acquisition of NMR spectra on a Bruker Ascend 500 MHz NMR spectrometer, the samples were dissolved in D_2_O and acetone was added as an internal reference (2.22 ppm).

### 2.7. Macromolecular Properties of Exopolysaccharides

#### 2.7.1. Intrinsic Viscosity

The intrinsic viscosity [*η*] [mL/mg] was determined in duplicate with a LOVIS rolling ball viscometer (Anton Paar GmbH, Ostfildern, Germany) as described previously [29]. The Huggins equation was used to calculate [*η*] of a solvent/polysaccharide pair on the basis of the dynamic viscosity of aqueous EPS solutions of different concentration.

#### 2.7.2. Molecular Mass

The molecular mass distribution of fEPS was estimated with a SECcurity² size exclusion chromatography system (PSS Polymer Standard Service, Mainz, Germany), equipped with an RI detector. One hundred µL of a pre-filtered (0.45 µm) sample (approx. 2 g/L) were injected, eluted with 50 mmol/L NaNO_3_/7.7 mmol/L NaN_3_ buffer and separated on three PSS Suprema columns (1 × 100 Å + 2 × 3000 Å). The weight average molecular mass *M_W_* [Da] and numeric average molecular mass *M_N_* [Da] were calculated from a calibration curve with pullulan standards from PSS (342–2,560,000 Da). The polydispersity index *Đ* [-] is given by *M_W_*/*M_N_* [-]. Under the assumption of a spherical molecule, the hydrodynamic radius *R_H_* [nm] can be calculated from *M_W_* and [*η*] (*N_A_*: Avogadro constant) [30]:

(1) $$R_{H}=\sqrt[{3}]{\frac{3\cdot \left[\eta \right]\cdot M_{W}}{10\cdot \pi \cdot N_{A}}}\;$$

#### 2.7.3. Water Binding Capacity

The moisture load at defined *r.h.* levels was determined with a Q5000SA dynamic vapor sorption analyzer (TA Instruments, New Castle, DE, USA) at 25 °C, following the procedure described previously [29,31] with some modifications. Approx. 5 mg sample, pre-dried over P_2_O_5_, was weighed into a quartz crucible and exposed to a relative humidity of 0% in the measuring chamber until constant mass (mass difference ≤ 0.1 g/kg within 5 min) was achieved. Relative humidity was then increased gradually to 98% (increments of 10%, last increment: 8%). The initial mass at each humidity level was denoted as *m_0_*, and abort criterions at each *r.h.* step were either a constant mass *m* or a running time of ≥1500 min. The final mass at each humidity level *m_∞_* was used to calculate the equilibrium moisture loads *X_r.h._* (g H_2_O/g dry matter) and to generate moisture sorption isotherms. For desorption isotherms, humidity was decreased in the same increments. The sorption isotherms were then fitted with the Guggenheim–Anderson–De Boer (*GAB*) equation that is valid between 0% ≤ *r.h.* ≤ 80% [9]:

(2) $$X=\frac{X_{0}\cdot C_{GAB}\cdot k_{GAB}\cdot a_{W}}{\left(1-k_{GAB}\cdot a_{W}\right)\cdot \left(1-k_{GAB}\cdot a_{W}+C_{GAB}\cdot k_{GAB}\cdot a_{W}\right)}$$

*X*_0_ [g/g] represents the water monolayer coverage and *k_GAB_* [-] and *C_GAB_* [-] are equation constants.

As none of the sorption kinetic models introduced in the literature describes the sorption phenomena physically in a sufficient way, simple first order kinetics were fitted. The function type is based on the parallel exponential kinetics model [11], but simplified by using only one function instead of two. The time-dependent mass *m(t)* of each sorption step was fitted separately to

(3) $$m\left(t\right){=m}_{0}{+m}_{\infty }\cdot \left(1-e^{-\frac{t}{k}}\right)$$

with *k* [min] representing the time when (1 − e^−1^)∙*M_∞_* ≈ 0.63∙*M_∞_* was adsorbed on each *r.h.* level. Due to the geometry of the crucible, moisture sorption is not independent from sample mass. Thus, sorption time *τ* = *k*/*m*_0*(r.h. =* 0*%)*_ [min/mg] was used to estimate moisture sorption velocity.

### 2.8. Statistics

Unless stated otherwise, data are expressed as arithmetic mean ± half deviation range (*n* = 2) or arithmetic mean ± standard deviation (*n* > 2).

## 3. Results and Discussion

### 3.1. Microbial Production of Free and Capsular Exopolysaccharides

Cultivations of *S. thermophilus* ST-1G and ST-2E were stopped at the end of the exponential growth phase (after 15 h and 10 h, respectively). For ST-1G, a significantly higher cEPS concentration of 348 mg GE/L was observed than for ST-2E (61 mg GE/L), whereas tEPS concentration was similar for both strains (Table 1). The yields *Y_EPS/X_* were also similar, but *Y_cEPS/X_* was significantly higher for ST-1G. As published research mainly focused on fEPS, data on cEPS concentrations and yields are scarce. Thus, we recalculated these parameters for EPS producing *S. thermophilus* strains from data on biomass production and cEPS and fEPS amounts published in previous studies [3,24,27]: *Y_cEPS/X_* (and cEPS fractions) were 13 mg/g (13% of tEPS), 17 mg/g (13% of tEPS) and 169 mg/g (38% of tEPS) for DGCC7710, DGCC7919 and ST143+, respectively. According to these findings, it is possible to distinguish between *S. thermophilus* strains that produce high cEPS amounts (ST-1G, ST143+), and strains with low cEPS fractions (ST-2E, DGCC7710, DGCC7919). On the basis of the Wzx/Wzy dependent synthesis pathway, it is assumed that cEPS of *S. thermophilus* exhibit the same chemical structure as the respective fEPS [1,32]. After assembling the repeating unit inside the bacterial cell through the action of different glycosyl transferases, the building blocks are translocated to the outside of the cell by Wzx flippase. The polymerization to the final macromolecule by Wzy polymerase [33] results in cEPS present at the bacterial cell surface. In Gram positive bacteria such as *S. thermophilus*, fEPS are released into the fermentation medium by the action of a single enzyme which cleaves the covalent bond between polysaccharide and cell membrane [34,35]. However, the conditions for this release which affects the final molecular mass of the EPS as well as the fractions of cEPS and fEPS are still unclear [1]. It may be assumed that strains with a high fraction of cEPS show a lower enzymatic activity for EPS release than strains with a lower cEPS fraction.

**Figure 1:**
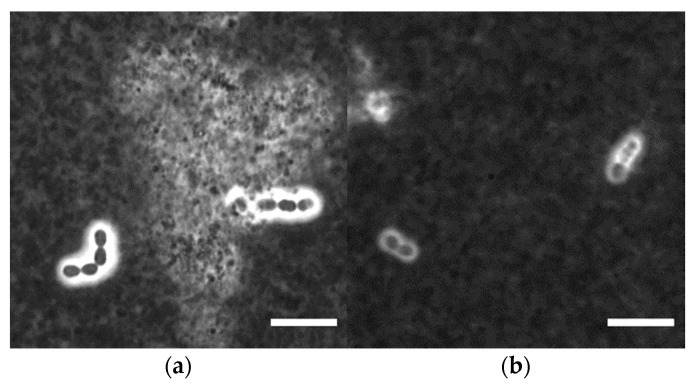
Light micrographs of stirred milk gels fermented with *S. thermophilus* (**a**) ST-1G or (**b**) ST-2E after dilution with deionized water and negative staining with Indian ink. Light areas around the cells indicate capsular EPS, in the background are ink and gel particles. Scale bar: 10 µm.

The quantitative difference in cEPS fractions can also be visualized by light microscopy (Figure 1). ST-1G showed a thicker cEPS layer in fermented milk than ST-2E. By using confocal laser scanning microscopy, Hassan et al. [4] observed that some non-ropy EPS producing strains had a capsule diameter in milk that was twice as high as that for ropy EPS producers.

**Figure 2:**
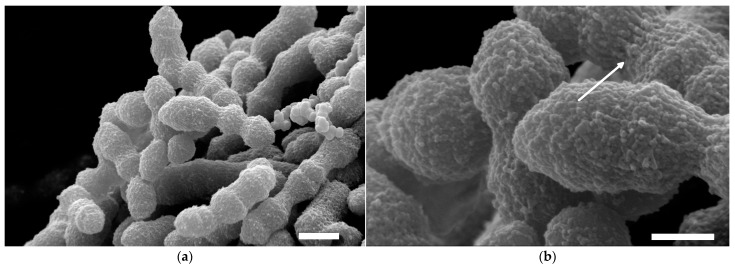
SEM micrographs of *S. thermophilus* ST-1G cells with attached cEPS. (**a**) Overview, scale bar: 1 µm; (**b**) detailed view, scale bar: 500 nm. Arrow indicates cEPS strands.

The SEM micrographs showed that the ellipsoid-shaped ST-1G cells are surrounded by a nano-sized bubble-like network cover of cEPS causing a rough, rugged surface that holds several cells together in larger units with a rope-like structure (Figure 2a). The cEPS are tightly bound to the cell walls in intricate strands (Figure 2b, arrow). Similar structures were found for other bacteria, for instance *Escherichia coli* or *Klebsiella pneumonia* [36,37]. The cEPS layer around the ST-1G cells can be confirmed by the shortest diameter of the ellipsoid (cells + cEPS) of 0.84 ± 0.05 µm which is comparable to the cell diameter of ST143+ (0.84 ± 0.04 µm) determined in a former study [3], while the mutant ST143− without cEPS was smaller (0.75 ± 0.10 µm).

In our recent study, susceptibility to syneresis was lower for stirred skim milk gels with ST-1G EPS than for milk acidified with ST-2E EPS [23]. For set skim milk gels, Hassan et al. [6] found a higher serum retention with cEPS of a capsule diameter of ~5 μm compared with a capsule diameter of ~2 μm. Thus, thick capsules and a high fraction of cEPS predominantly reduce syneresis of fermented milk.

### 3.2. Exopolysaccharide Structure

As fEPS result from the cleavage of cEPS from the cell wall, the same structural elements are usually present in cEPS and fEPS; this was previously shown for fEPS and cEPS from *S. thermophilus* DGCC7710 [27]. However, the isolation of fEPS is much more straightforward and usually results in acceptable yields and purity. As a consequence, we analyzed fEPS to obtain information on the structural composition of the EPS formed by the two strains.

Monosaccharide composition and the results of glycosidic linkage analysis are shown in Table 2. Both fEPS contain comparable portions of galactose and glucosamine while the portions of glucose and rhamnose clearly differ: ST-1G fEPS contain more glucose but less rhamnose than ST-2E fEPS. Differences between the two samples are also evident from the methylation analysis results. Varying portions of glycosidic linkages were detected for rhamnose which represented linear (1,2- and, in case of ST-2E, 1,3-linkages) and branched (1,2,3- and 1,2,3,4-linkages) rhamnose units. Notably, PMAAs corresponding to branched rhamnose units were of higher relative abundance in ST-2E fEPS than in ST-1G, which also contained no 1,3-linked rhamnose units. However, glucose-derived glycosidic linkages were much more complex for EPS from ST-1G, which contained 1,3-, 1,4-, 1,6-, and 1,3,6-linked glucose units in addition to terminal glucose. In contrast, ST-2E only contained 1,3- and 1,3,6-linked glucose units (besides terminal glucose). While no galactose PMAAs were detected in ST-1G fEPS, ST-2E fEPS contained terminal galactopyranose and galactofuranose. These glycosidic linkages as well as 1,3- and 1,3,6-linked glucose also appear in the fEPS from *S. thermophilus* DGCC7710 [29]; thus, this EPS or a similar one may be present. Overall, the two fEPS preparations seem to contain polysaccharides with a clearly different structure. To gain more information on the EPS structure, NMR spectroscopy was used. However, only proton spectra were acquired for the two EPS (Figures S1 and S2); it was not possible to record meaningful two-dimensional spectra due to the low purity of the samples and, most likely, due to the fast T2 relaxation of the polysaccharides. The proton spectrum of ST-2E was of low quality and only contained a few low-abundance signals. Nevertheless, it was possible to identify three anomeric signals which were also observed in the proton spectrum of *S. thermophilus* DGCC7710 EPS [29]. Therefore, methylation analysis as well as NMR spectroscopy indicate that the same or a very similar EPS is present. In contrast, rhamnose-derived signals could not be identified, so that it is possible that a mixture of two different EPS is present. ST-1G yielded a very complex spectrum with multiple carbohydrate-derived signals and impurities which did not allow further assignments. However, the data from the structural analyses demonstrate that ST-1G and ST-2E produce complex EPS or EPS mixtures which have clearly different molecular structures.

### 3.3. Macromolecular Properties of Free Exopolysaccharides

The molecular mass, intrinsic viscosity and hydrodynamic radius were higher for ST-2E fEPS than ST-1G fEPS (Table 3). As a consequence of the higher *M_W_*, fEPS from ST-2E also showed a more polydisperse molecular mass distribution, a typical trend for biomacromolecules.

In a study with two EPS from *S. thermophilus* which had the same repeating unit but differed in their molecular mass, Faber et al. [39] observed ropiness in fermented milk only when EPS with higher molecular mass were present. Surber et al. [23] observed that fEPS from ST-2E but not fEPS from ST-1G induced ropy behavior in fermented milk; this also applies to ST-1G and ST-2E fEPS, irrespective of differences in chemical structure. A further reason for the absence of a ropy phenotype of ST-1G may be its high cEPS and its low fEPS fraction.

Differences in texture properties can be related to structural and macromolecular properties, e.g., a higher product viscosity of ST-2E [23] and other strains [8,39,40] correlated positively with enhanced macromolecular properties of fEPS. Compared to fEPS from ST-2E, fEPS from ST-1G contained more branched glucose units but less branched rhamnose units and had a lower intrinsic viscosity. Hence, less interaction of the EPS with proteins or among themselves is possible. This might be responsible for the higher particle size and the lower shear viscosity of milk fermented with ST-1G [23]. In another study, we observed that fEPS from ST-143+ or DGCC7710 have a comparable intrinsic viscosity, but that ST-143 fEPS are β(1→4)-linked, have dimeric side chains and therefore probably a stiffer backbone than the DGCC7710 fEPS [29,41]. Stirred skim milk gels with ST-143+ showed a lower shear viscosity than those with DGCC7710 [8]. Thus, the stiffer backbone can be relevant for the reduced structural regeneration after shearing. However, this is in contrast to other *S. thermophilus* and *Lactobacillus delbrueckii* ssp. *bulgaricus* strains [42,43] for which a stiffer backbone was associated with an increased shear viscosity of fermented milk. General statements on the structure-function relationship are, however, difficult due to differences in more than one structural property. For ongoing research, higher EPS yields and isolate purities are needed.

### 3.4. Sorption Behaviour of Exopolysaccharides at Equilibrium State

**Figure 3:**
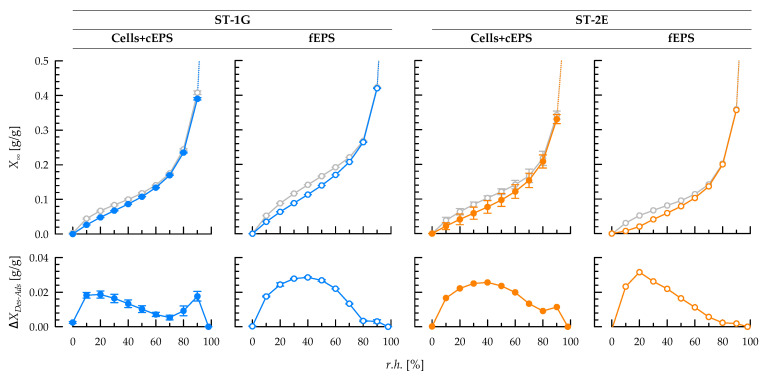
(**Top**): Adsorption (color) and desorption (grey) isotherms of fEPS (open symbols) and cell isolates with attached cEPS (closed symbols) from *S. thermophilus* ST-1G (blue) and ST-2E (orange). (**Bottom**): Difference between desorption and adsorption isotherms. *r.h.*: relative humidity; *X_∞_*: equilibrium moisture load; *X_Des-Ads_*: difference in moisture load between desorption and adsorption.

Generally, moisture sorption of freeze-dried cells with attached cEPS and the corresponding fEPS increased continuously with *r.h.*, and the sorption isotherms showed the typical sigmoidal shape of type II isotherms according to Brunauer et al. [44] (Figure 3). For between-sample comparison, the absolute moisture load after *r.h.* equilibration, *X*_90_ at *r.h.* = 90% (adsorption) was used (Table 4). *X*_90_ was higher for ST-1G isolates (fEPS as well as for cells with cEPS; difference: approx. 0.06 g/g) than for ST-2E isolates. Thus, moisture sorption may primarily depend on EPS structure. In a previous study with the cEPS-producing *S. thermophilus* DGCC7710, it was shown that cells with cEPS, isolated cEPS and fEPS had a similar *X*_90_, but the lack of the cEPS layer decreased *X*_90_ of the bacterial cells by approx. 0.1 g/g [27]. Thus, cEPS attached to the cell wall are crucial for moisture adsorption. This was confirmed in the present study for both strains, despite the relatively low fraction of cEPS in the cell isolates (7.5 and 1.7% for ST-1G and ST-2E, respectively): *X*_90_ was only slightly higher (approx. 0.03 g/g) for fEPS than for cells with cEPS. Without a cEPS layer, a much lower *X*_90_ for bacterial cells would be expected.

Higher moisture loads of cells with cEPS or fEPS may contribute to lower syneresis of stirred fermented milk. In centrifugation tests for syneresis estimation, the serum retention was higher when ST-1G was used for milk fermentation [23]. Mende et al. [8] also observed lower syneresis of stirred gels with non-ropy fEPS and cEPS from DSM8713 than with ropy and cEPS from DGCC7710 or ST-143. Gentès et al. [42] reported a lower syneresis with ropy fEPS than with non-ropy fEPS; however, they did not report on their ability to produce cEPS.

The sorption isotherms formed hysteresis areas with an increased difference between desorption and adsorption especially at low *r.h.* (Figure 3), and the absolute hysteresis area was significantly higher for ST-2E cells than ST-1G cells (Table 4). An increased hysteresis area may serve as indicator for a more pronounced water binding and for the potential of structural and conformational rearrangements, and more energy is needed to remove the water during desorption. A hysteresis at low *r.h.* is typical for porous samples that possess energy-rich binding sites with a high surface energy [45,46]. As ST-1G cells are covered by a thick cEPS layer, a higher fraction of water may be bound in this polysaccharide than inside the bacterial cell compared to ST-2E, and water can desorb more easily from the porous cEPS layer than from the inside of the bacterial cell, resulting in a lower hysteresis. This is in line with a previous study with *S. thermophilus* ST143+ (cells with cEPS) and ST143− (cells without cEPS), where ST143− cells showed a higher hysteresis than ST143+ cells [3]. However, the hysteresis area was higher for ST-1G fEPS than for ST-2E fEPS. This may be explained by structural differences between both EPS that are not dominating when exploring bacterial cells with a different thickness of cEPS layers around the cells.

The isotherms between 0% ≤ *r.h.* ≤ 80% were fitted with the Guggenheim–Anderson–De Boer equation (Table 4). For all sample fits, r² was ≥0.99. The equation parameters may provide additional information on moisture sorption behavior: (I) At the beginning of moisture adsorption, the sample is covered with a single monolayer of water (*X*_0_). *X*_0_ was between 0.057–0.066 g/g for all samples, except for fEPS from ST-1G which had a significantly higher *X*_0_. In a previous study, lower *X*_0_ were observed for cell isolates with cEPS compared to cells without a polysaccharide layer, but *X*_0_ remained unaltered after removal of the cEPS layer [3]. (II) The constant *C_GAB_*, which reflects the binding strength of further water layers on top of the monolayer, was lower for cell isolates than for fEPS isolates during adsorption. Generally, higher *C_GAB_* during desorption can be interpreted with a higher energy that is necessary to remove bound water layers from the sample instead of binding new water layers which consequently leads to the occurrence of a hysteresis area. (III) *k_GAB_* represents the difference of the chemical potential of bound water in the multilayer, compared to free water (*k_GAB_* = 1): lower *k_GAB_* values can be explained by enhanced interactions between water and sample surface. This applies to ST-2E cells, compared to ST-1G cells, presumably due to the higher fraction of water that is bound inside the bacterial cell and not in the polysaccharide layer. For cEPS-negative ST143− cells, stronger interactions between water and sample occurred and *k_GAB_* was lower compared to cEPS-positive ST143+ cells [3]. As already observed for the hysteresis areas, the effect of bacterial cells and the thickness of the polysaccharide layer covers the influence of the different chemical structure of ST-1G and ST-2E. Consequently, the *k_GAB_* constants for both fEPS showed a different trend compared to the respective cell isolates.

### 3.5. Sorption Kinetics of Exopolysaccharides

**Figure 4:**
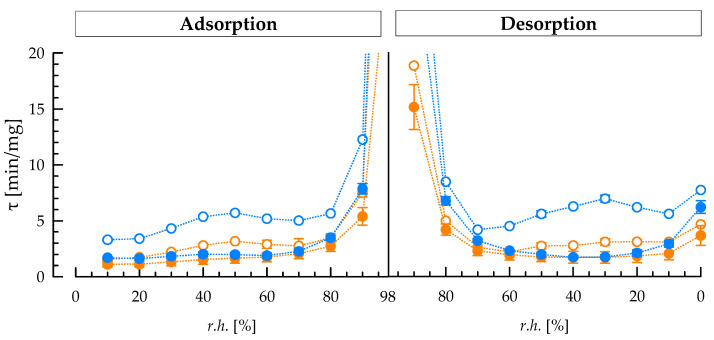
Sorption time *τ* of *S. thermophilus* ST-1G (blue) and ST-2E (orange) cell isolates with attached cEPS (closed symbols) and fEPS (open symbols) as a function of relative humidity *r.h.* during adsorption and desorption.

To quantify the sorption velocity, *τ* was determined for each *r.h.* level during adsorption and desorption (Figure 4). During adsorption, *τ* is nearly constant until *r.h.* = 80% is reached, i.e., the time to adsorb approx. two third of *M_∞_* on each *r.h.* level does not differ. At *r.h.* > 80%, not only more water was adsorbed absolutely, but also *τ* increased for all samples. Cell isolates with attached cEPS adsorbed faster, i.e., showed lower *τ*, than the respective fEPS, independent of *r.h.* The difference between cell and fEPS isolates was significantly higher for ST-1G (e.g., 4.4 min/mg at *r.h.* = 90% during adsorption) than for ST-2E (2.1 min/mg). As regards the two cell isolates, ST-2E cells adsorb faster (2.5 min/mg at *r.h.* = 90%) than ST-1G cells, despite their lower *k_GAB_* and thus stronger water binding in the multilayer. It is assumed that the thickness of the cEPS layer reduces the sorption velocity for ST-1G cells and that it covers the reverse effect that was observed for fEPS, where ST-2E fEPS adsorb faster (4.8 min/mg at *r.h.* = 90%) than ST-1G fEPS.

During desorption, similar trends were observed as during adsorption. Because more energy is needed to remove all remaining water at low relative humidity, *τ* values were higher at *r.h.* = 0% compared to *r.h.* = 10% for all samples. As ST-2E fEPS were able to adsorb moisture faster than ST-1G fEPS, desorption was also faster (e.g., by 3.5 min/mg at *r.h.* = 80%). This could be a reason for the higher sedimentation velocity of gel particles in stirred ST-2E gels that was observed during phase separation in an analytical photo-centrifuge [23]. Because of the higher *τ* and lower sedimentation velocity, ST-1G contributes to slower phase separation during cold storage of stirred gels.

## 4. Conclusions

*S. thermophilus* ST-1G and ST-2E produced cEPS and fEPS with a different degree of ropiness: ST-2E secretes ropy fEPS, whereas the fEPS of ST-1G are non-ropy. The strains also differed in their cEPS fraction. As observed by light and scanning electron microscopy, capsules of ST-1G (cEPS fraction: 53%) were thicker than the capsules of ST-2E (cEPS fraction: 10%). Analyses of the fEPS revealed structural differences: fEPS from ST-1G had more branched glucose units, whereas fEPS from ST-2E had more branched rhamnose units. In line with a higher molecular mass, this resulted in a higher intrinsic viscosity for fEPS from ST-2E. Higher intrinsic viscosity is often related to ropy fEPS. Thus, the ropy character when ST-2E fEPS are present may be traced back to its macromolecular and structural features as well as the higher fEPS fraction (90%) compared to ST-1G (47%).

To understand syneresis phenomena in fermented milk, isolates of cEPS attached to cells and fEPS were subjected to moisture adsorption and desorption experiments. The high cEPS fraction of ST-1G led to higher equilibrium moisture loads at constant relative humidity, lower hysteresis between adsorption and desorption isotherms and thus a lower chemical potential of the bound water, compared to cells with attached cEPS from ST-2E. This correlated well with a lower syneresis of fermented milk produced with ST-1G. Additionally, the sorption velocity of EPS from *S. thermophilus* was estimated for the first time. Cells with attached cEPS adsorb moisture faster than the fEPS from the same strain, and the slower adsorption and desorption of ST-1G compared to ST-2E isolates may be also related to the lower syneresis of fermented milk. Therefore, strains with a high fraction of cEPS could be of interest in future strain selections because of their ability to limit syneresis without inducing ropiness.

## Figures and Tables

**Table 1:** EPS amount (*n* = 6), biomass (*n* = 3) and yield of enriched whey permeate media fermented with *S. thermophilus* ST-1G or ST-2E. Values are shown as means ± standard deviation ^1^.

Parameter	ST-1G	ST-2E
EPS amount		
tEPS [mg GE/L]	659 ± 14	601 ± 67
fEPS [mg GE/L] (% of tEPS)	311 ± 29 (47%)	540 ± 12 (90%)
cEPS [mg GE/L] (% of tEPS)	348 (53%)	61 (10%)
Biomass and Yield		
X [g/L]	4.31 ± 0.13	3.65 ± 0.06
*Y_EPS/X_* [mg/g]	153	165
*Y_cEPS/X_* [mg/g]	75	17

^1^ Abbreviations are: fEPS: free exopolysaccharides; tEPS: total EPS; cEPS: capsular EPS, cEPS = tEPS − fEPS; GE: glucose equivalent; *X*: biomass; *Y_EPS/X_*: yield (EPS per biomass); *Y_cEPS/X_*: yield (cEPS per biomass).

**Table 2:** Monosaccharides (mol%, determined by HPAEC-PAD after TFA hydrolysis) and glycosidic linkages (mol%, determined by methylation analysis) in fEPS from ST-1G and ST-2E ^1^.

Monosaccharides	ST-1G fEPS	ST-2E fEPS	Glycosidic Linkages	ST-1G fEPS	ST-2E fEPS
Glucose	36.5	15.7	t-Glc*p*	21.0	21.0
			1,3-Glc*p*	6.2	4.1
			1,4-Glc*p*	2.1	-
			1,3,6-Glc*p*	7.6	3.1
			1,6-Glc*p*	4.2	-
Galactose	2.8	3.7	t-Gal*f*	-	1.0
			t-Gal*p*	-	9.4
Rhamnose	43.8	64.1	1,2-Rha*p*	37.8	15.3
			1,2,3-Rha*p*	8.3	15.8
			1,2,3,4-Rha*p*	12.8	22.1
			1,3-Rha*p*	-	8.4
Glucosamine, N-Acetyl glucosamine	16.9	16.5	1,3-GlcN*p*	-	- ^2^
			1,4-GlcN*p*	- ^2^	- ^2^

^1^ Abbreviations are: Glc, glucose; Gal, galactose; Rha, rhamnose; GlcN, glucosamine; *p*, pyranose; *f*, furanose. ^2^ The corresponding PMAAs were only detected in low amounts (most likely due to incomplete acid hydrolysis [38]); thus data are not included.

**Table 3:** Weight average molecular mass *M_W_*, polydispersity index *Đ*, intrinsic viscosity [*η*] and hydrodynamic radius *R_H_* of free EPS isolates (fEPS) from *S. thermophilus* ST-1G or ST-2E (*n* = 2).

Parameter	ST-1G fEPS	ST-2E fEPS
*M_W_* [10^6^ Da]	5.90 ± 0.11	12.66 ± 0.33
*Đ* [-]	5.0 ± 0.1	10.3 ± 0.4
[*η*] [mL/mg]	0.250 ± 0.012	0.269 ± 0.001
*R_H_* [µm]	61.5 ± 1.0	81.4 ± 0.1

**Table 4:** Moisture load at *r.h.* = 90% (*X*_90_), hysteresis area between adsorption and desorption isotherms and Guggenheim–Anderson–De Boer (GAB) equation parameters *X*_0_, *k_GAB_* and *C_GAB_* for adsorption and desorption.

	ST-1G		ST-2E	
	Cells + cEPS	fEPS	Cells + cEPS	fEPS
*X*_90_ [g H_2_O/g dry matter]	0.390 ± 0.003	0.420 ± 0.001	0.331 ± 0.013	0.357 ± 0.010
Hysteresis area [area units]	1.155 ± 0.069	1.667 ± 0.009	1.469 ± 0.079	1.394 ± 0.080
GAB model parameters (adsorption)				
*X*_0_ [g/g]	0.064 ± 0.001	0.083 ± 0.001	0.066 ± 0.003	0.057 ± 0.003
*C_GAB_* [-]	7.645 ± 0.188	8.519 ± 0.037	7.039 ± 0.073	8.441 ± 0.137
*k_GAB_* [-]	0.934 ± 0.002	0.894 ± 0.001	0.899 ± 0.002	0.947 ± 0.004
GAB model parameters (desorption)				
*X*_0_ [g/g]	0.063 ± 0.001	0.094 ± 0.001	0.071 ± 0.003	0.056 ± 0.006
*C_GAB_* [-]	25.163 ± 3.276	17.219 ± 0.284	16.834 ± 0.783	33.789 ± 12.919
*k_GAB_* [-]	0.941 ± 0.004	0.862 ± 0.001	0.883 ± 0.005	0.948 ± 0.010

## Data Availability

The datasets generated for this study are available on request to the corresponding author.

## Supplementary Materials

The following supporting information can be downloaded at: https://www.mdpi.com/article/10.3390/foods12030596/s1, Figure S1: ^1^H-NMR spectrum of fEPS from ST-1G.; Figure S2: ^1^H-NMR spectrum of fEPS from ST-2E. The signal annotation in the anomeric region was derived by comparison with a previously published proton spectrum of *Streptococcus thermophilus* EPS ([29]).
