# Supplementary material for: Capsular Exopolysaccharides from Two Streptococcus thermophilus Strains Differ in Their Moisture Sorption Behavior
Source: Foods. 2023 Jan 31;12(3):596. doi: 10.3390/foods12030596 (PMC9914836; doi:10.3390/foods12030596)
Supplement: Supplementary file 1 [file foods-12-00596-s001.zip › foods-2160960-supplementary.pdf]

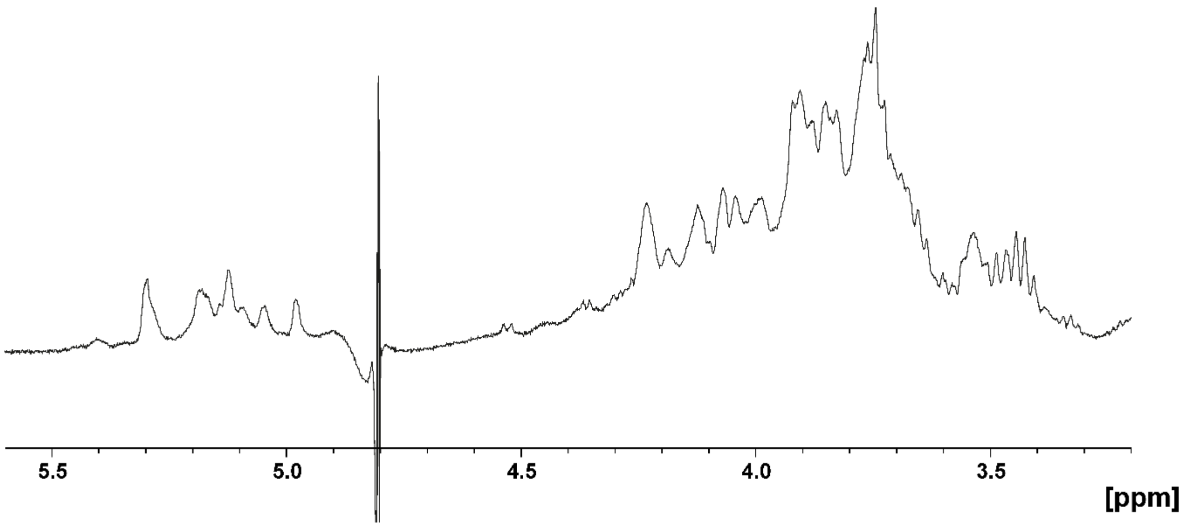

Figure S1: ^{1}H-NMR spectrum of fEPS from ST-1G.

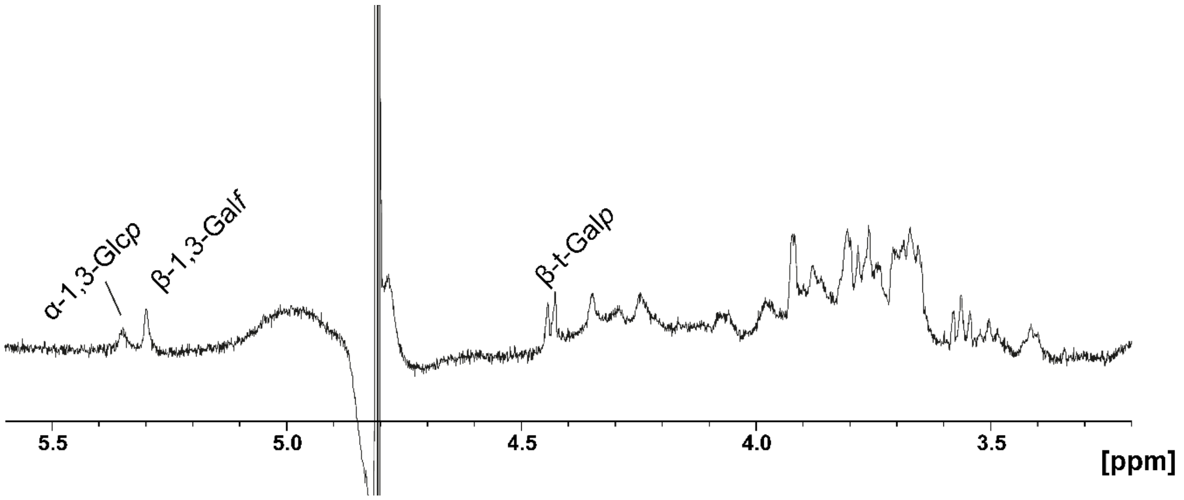

Figure S2: ^{1}H-NMR spectrum of fEPS from ST-2E. The signal annotation in the anomeric region was derived by comparison with a previously published proton spectrum of *Streptococcus thermophilus* EPS (Nachtigall et al. *Food Hydrocolloids* 2019, 97, 105181).
